# The Protective Role of the TOPK/PBK Pathway in Myocardial Ischemia/Reperfusion and H_2_O_2_-Induced Injury in H9C2 Cardiomyocytes

**DOI:** 10.3390/ijms17030267

**Published:** 2016-02-23

**Authors:** Guozhe Sun, Ning Ye, Dongxue Dai, Yintao Chen, Chao Li, Yingxian Sun

**Affiliations:** 1 Department of Cardiovascular Medicine, the First Hospital of China Medical University, Shenyang 110001, Liaoning, China; gzhsun66@163.com (G.S.); yening_cmu@163.com (N.Y.); daidongxue253@126.com (D.D.); chenyintao1990@126.com (Y.C.); 2 Department of Biochemistry and Molecular Biology, China Medical University, Shenyang 110122, Liaoning, China; ydyzqy1@126.com

**Keywords:** ischemia/reperfusion, ischemic preconditioning, oxidative stress, TOPK/PBK

## Abstract

T-LAK-cell-originated protein kinase (TOPK) is a PDZ-binding kinase (PBK) that was recently identified as a novel member of the mitogen-activated protein kinase (MAPK) family. It has been shown to play an important role in many cellular functions. However, its role in cardiac function remains unclear. Thus, we have herein explored the biological function of TOPK in myocardial ischemia/reperfusion (I/R) and oxidative stress injury in H9C2 cardiomyocytes. I/R and ischemic preconditioning (IPC) were induced in rats by 3-hour reperfusion after 30-min occlusion of the left anterior descending coronary artery and by 3 cycles of 5-min I/R. Hydrogen peroxide (H_2_O_2_) was used to induce oxidative stress in H9C2 cardiomyocytes. TOPK expression was analyzed by western blotting, RT-PCR, immunohistochemical staining, and immunofluorescence imaging studies. The effects of TOPK gene overexpression and its inhibition via its inhibitor HI-TOPK-032 on cell viability and Bcl-2, Bax, ERK1/2, and p-ERK1/2 protein expression were analyzed by MTS assay and western blotting, respectively. The results showed that IPC alleviated myocardial I/R injury and induced TOPK activation. Furthermore, H_2_O_2_ induced TOPK phosphorylation in a time-dependent manner. Interestingly, TOPK inhibition aggravated the H_2_O_2_-induced oxidative stress injury in myocardiocytes, whereas overexpression relieved it. In addition, the ERK pathway was positively regulated by TOPK signaling. In conclusion, our results indicate that TOPK might mediate a novel survival signal in myocardial I/R, and that its effect on anti-oxidative stress involves the ERK signaling pathway.

## 1. Introduction

In patients with acute myocardial infarction (AMI), the most effective treatment strategy for reducing myocardial ischemic injury is timely and effective myocardial reperfusion. However, the process of reperfusion can cause additional cardiomyocyte dysfunction and death, which is generally referred to as myocardial reperfusion injury [1]. Some progress has been made in the past decade in relation to understanding the mechanisms of ischemia/reperfusion (I/R) injury and finding strategies to address it; however, the protective effects of clinical therapeutic approaches seem limited [2]. Thus, there remains an urgent need to understand additional molecular events that are activated by I/R, which eventually can be used as new therapeutic targets. Ischemic preconditioning (IPC) was first reported by Murry and associates [3] and has been exploited as a powerful endogenous mechanism for cardioprotection against I/R injury. However, no established therapeutic strategies have been devised based on IPC until now, and this might be due to inadequate understanding of the IPC. Therefore, future research and therapeutic strategies to prevent I/R injury must be planned based on the information derived from the detailed understanding of the IPC process.

Oxidative stress generated from mitochondrial reactive oxygen species (ROS) during reperfusion has long been considered as one of the major mechanisms in myocardial I/R injury [4]. IPC has the ability to alleviate the oxidative stress during reperfusion and protect against I/R injury [5]. Furthermore, extracellular signal-regulated kinase (ERK), which is activated by hydrogen peroxide (H_2_O_2_)-induced oxidative stress, plays an important role in protecting cardiomyocytes from apoptosis [6]. The anti-apoptotic protein Bcl2 and the pro-apoptotic protein Bax are the key signaling molecules involved in regulating myocardial apoptosis in response to I/R and oxidative damage of cardiomyocytes [7] and are downstream of ERK signaling. In our study, we tried to identify a novel protective signal associated with IPC, which might activate antioxidative pathways and protect the myocardium from oxidative stress injury.

T-LAK-cell-originated protein kinase (TOPK), a novel mitogen-activated protein kinase kinase (MAPKK)-like serine/threonine kinase, is a member of the MAPKK family and is involved in many cellular functions, including the promotion of tumor development, regulation of cell growth, and inhibition of apoptosis [8,9,10,11,12,13]. It is highly expressed in many organs during normal fetal development and also in proliferative malignant cells [14,15,16,17]. TOPK can prevent RPMI7951 melanoma cells from undergoing UVB-induced apoptosis through regulation of peroxidase activity and blocking intracellular H_2_O_2_ accumulation [12]. This suggests that TOPK has an ability to regulate oxidative stress. TOPK inhibition has also been shown to disrupt colon cancer cell growth by downregulating the ERK pathway and promoting colon cancer cell apoptosis [18]. However, Gaudet *et al.* [19], who first identified and characterized TOPK, has only reported its mRNA expression in the heart. Other than this, there have been no studies reporting TOPK expression and function in normal myocardium or heart disease. Thus, we investigated the biological function of TOPK in myocardial I/R and oxidative stress-induced injury of H9C2 cardiomyocytes.

## 2. Results

### 2.1. IPC Conferred Protection against Myocardial I/R Injury

**Figure 1:**
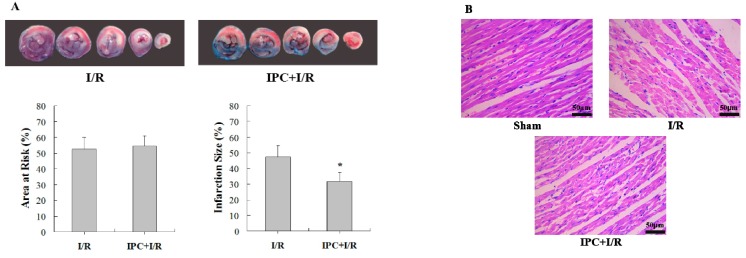

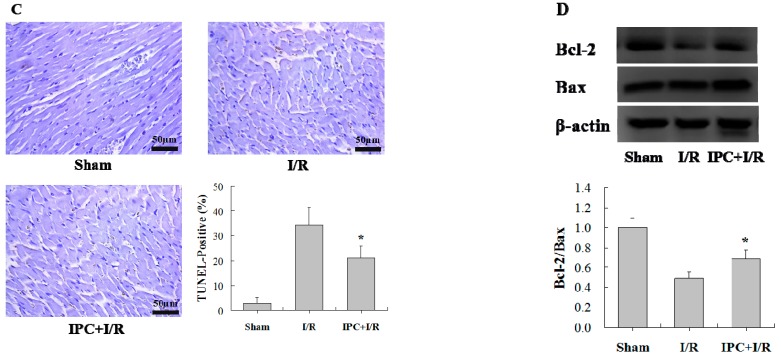
Ischemic preconditioning (IPC) treatment decreased myocardial ischemia/reperfusion (I/R) injury *in vivo*. (**A**) Representative photographs of Evans blue-TTC double staining and the quantitative analysis of myocardial infarct size between I/R (30-min ischemia/3-hour reperfusion) and IPC (three cycles of 5-min Ischemia/5-min Reperfusion)+I/R groups; (**B**) Hematoxylin and eosin (HE) staining of three groups, showing IPC mediated alleviation of the myocardium destruction; (**C**) Positive nuclear staining for myocardial apoptosis as assessed by the terminal deoxynucleotidyl transferase-mediated dUTP nick end labeling (TUNEL) assay and the quantitative analysis of apoptotic nuclei/total nuclei; (**D**) Analysis of Bcl-2 and Bax protein expression by western blotting and the corresponding quantitative analysis. β-actin was used as the loading control. *n* = 6. * *p* < 0.05 compared with I/R group.

To understand the role of IPC in myocardial I/R injury, rats were exposed to local I/R, IPC+I/R, or sham-operation. As a result, IPC decreased the infarct size compared to that in the I/R group (Figure 1A). Consistent with its effect on infarct size, IPC also relieved myocardial structural damage (Figure 1B) and reduced the rate of apoptosis (Figure 1C). Furthermore, IPC upregulated the ratio of BCL-2/BAX, as detected by western blotting, which then regulated cardiomyocyte apoptosis (Figure 1D).

### 2.2. IPC Activated TOPK Signaling Pathway

**Figure 2:**
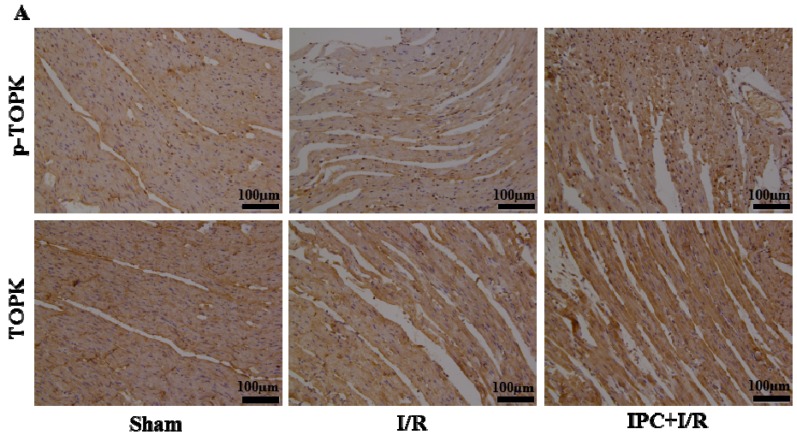

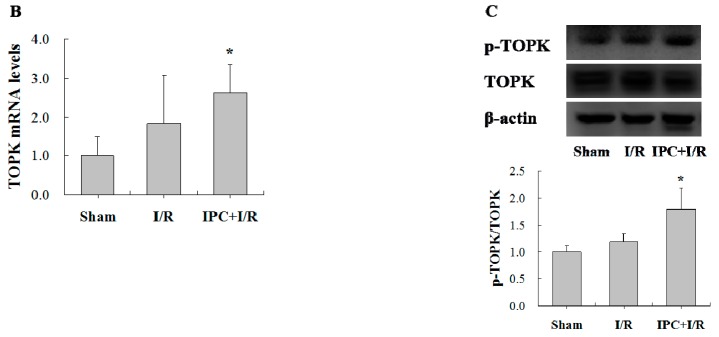
Ischemic preconditioning (IPC) treatment induced upregulation of TOPK in the rat myocardium after 30 min ischemia/3 h reperfusion. (**A**) Representative immunohistochemical staining of TOPK and p-TOPK in the ischemic area of left ventricular (LV) myocardial sections of rats subjected to sham operation, I/R, or IPC+I/R; (**B**) Detection of mRNA levels of TOPK by RT-PCR. GAPDH was used as the loading control; (**C**) Detection and quantitative analysis of TOPK and p-TOPK levels by western blotting. β-actin was used as the loading control. *n* = 6. * *p* < 0.05 compared with sham-operation group.

To explore the expression of TOPK in normal heart tissue and myocardium subjected to I/R injury and determine whether TOPK was significantly activated by IPC, we analyzed its expression in heart tissue following sham-operation, I/R, and IPC+I/R treatments by RT-PCR, western blotting, and immunohistochemistry (Figure 2). TOPK was observed to be significantly activated in the I/R and IPC+I/R groups, more so in the latter group. The active form p-TOPK, which was triggered by IPC and I/R, was mainly located in the nucleus. These results suggest that IPC induced protection against I/R injury, and this was accompanied by activation of the TOPK signaling pathway. Based on these results, we speculated that the activation of TOPK played a protective role against myocardial I/R injury.

### 2.3. H_2_O_2_ Activates TOPK in Cardiomyocytes in Time-Dependent Manner

**Figure 3:**
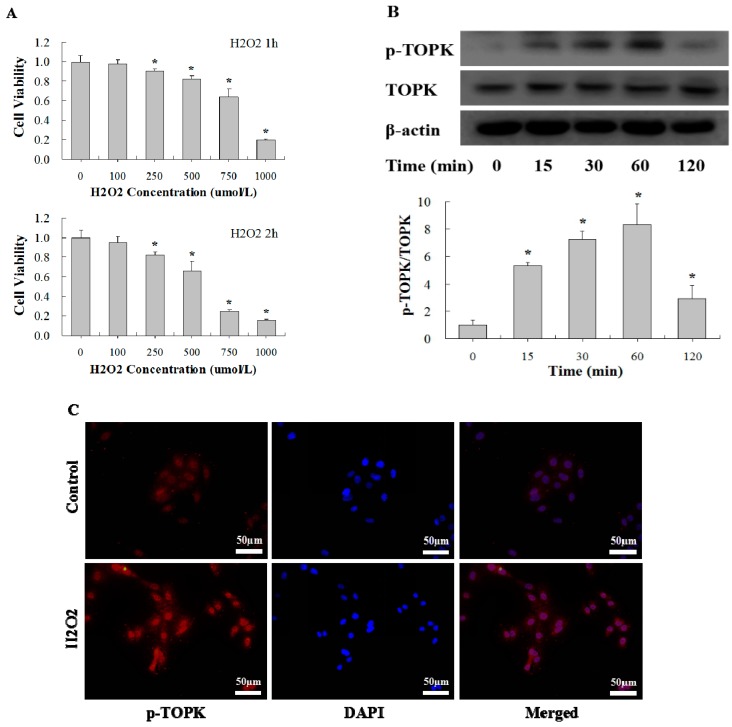
H_2_O_2_ treatment induced upregulation of p-TOPK in H9C2 cardiomyocytes. (**A**) Cultured H9C2 cardiomyocytes were stimulated with a different H_2_O_2_ concentrations (0–1000 µM) for various time points (1 or 2 h), and cell viability was evaluated by MTS assay; (**B**) Western blot analysis of TOPK activation by H_2_O_2_ treatment (750 µM for 0, 15, 30, 60, or 120 min); (**C**) Immunofluorescence staining of H9C2 cardiomyocytes with p-TOPK antibody (red) and DAPI for nuclei (blue) after treatment with 750 µM of H_2_O_2_ for 1 h. * *p* < 0.05 compared with nonstimulated controls.

As it has been well established that oxidative stress is one of the major mechanisms of I/R injury, we analyzed the role of TOPK in H_2_O_2_-induced oxidative stress injury in H9C2 cardiomyocytes. To determine the optimum concentration of H_2_O_2_ for induction of oxidative stress in cardiomyocytes, a series of H_2_O_2_ concentrations (0–1000 µM) were used for different time points (1 or 2 h) (Figure 3A). We observed that H_2_O_2_ reduced the cell viability of H9C2 cardiomyocytes in a concentration and time-dependent manner. Finally, incubation with 750 µM of H_2_O_2_ for 1 h was selected to induce oxidative stress injury in subsequent experiments. To determine the effect of oxidative stress on the activation of TOPK in cultured H9C2 cardiomyocytes, cells were incubated with 750 µM of H_2_O_2_ for different periods of time (0, 15, 30, 60, or 120 min). Western blot analysis showed that TOPK expression was markedly activated after 15–60 min of treatment, and the activation was rapid and transient. TOPK phosphorylation increased from 15 min, peaked at 30–60 min, and returned to the basal level at 120 min after the addition of H_2_O_2_ (Figure 3B). Immunofluorescence imaging analysis was also conducted to quantify changes in the level and localization of active p-TOPK in H9C2 cardiomycytes in response to H_2_O_2_ treatment (Figure 3C). Consistent with the results of immunohistochemistry analysis of heart tissue, p-TOPK was mainly located in the nucleus of normal H9C2 cardiomyocytes. Interestingly, p-TOPK signaling was significantly increased after incubation with H_2_O_2_ for 60 min.

### 2.4. TOPK Inhibitor Aggravated the Oxidative Stress Injury in H9C2 Cardiomyocytes via the ERK Signaling Pathway

**Figure 4:**
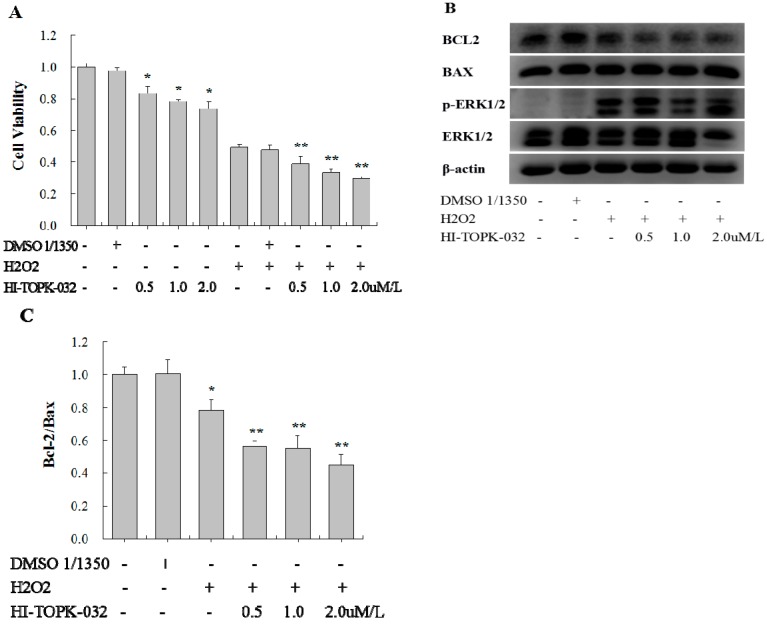
Analysis of the effects of the TOPK specific inhibitor HI-TOPK-032 on cell viability, apoptosis, and protein expression. H9C2 cardiomyocytes were incubated with HI-TOPK-032 at concentrations of 0.5, 1.0, and 2.0 µM for 24 h and then stimulated with 750 µM of H_2_O_2_ for 1 h. Dimethyl sulfoxide (DMSO) was used as a solvent control. (**A**) Cell viability as detected by MTS assay; (**B**) Western blot analysis of Bcl-2, Bax, ERK, and p-ERK expression. β-actin was used as a loading control; (**C**) The Bcl-2/Bax ratio was calculated and compared. * *p* < 0.05 compared with nonstimulated controls; ** *p* < 0.05 compared with H_2_O_2_-stimulated controls.

To determine the effect of H_2_O_2_-induced activated TOPK on the injury of H9C2 cardiomyocytes, we used a specific TOPK inhibitor, HI-TOPK-032, at concentrations of 0.5, 1.0, and 2.0 µM to inhibit its function. After 24 h in culture with or without HI-TOPK-032, the cells were further incubated with H_2_O_2_ (750 µM) for 1 h to induce oxidative stress injury. An equivalent concentration of dimethyl sulfoxide (DMSO) was used to treat cells as a solvent control. The cell viability of H9C2 cardiomyocytes was determined by MTS assay. HI-TOPK-032 treatment decreased both the basal cell viability and the cell viability following H_2_O_2_-induced oxidative stress injury in a concentration-dependent manner (Figure 4A). DMSO treatment had no significant effect on the cell viability of cardiomyocytes.

It has been demonstrated that the BCL-2/Bax ratio plays an important role in regulating cardiomyocyte apoptosis. Thus, we determined the activity of the BCL-2/Bax signaling pathway in H9C2 cardiomyocytes stimulated with H_2_O_2_ with or without HI-TOPK-032 to explore the role of TOPK in the apoptosis regulatory pathway. We observed that H_2_O_2_ decreased the ratio of BCL-2/Bax, and HI-TOPK-032 further inhibited the activity of BCL-2/Bax (Figure 4B,C). DMSO had no significant effect. Therefore, it appeared that HI-TOPK-032 might aggravate the H_2_O_2_–induced injury by further inhibiting the BCL-2/Bax signaling pathway.

In further exploring the signaling pathway that mediates the effect of HI-TOPK-032 on the BCL-2/Bax ratio, we identified that ERK signaling is activated in H9C2 cardiomyocytes stimulated with H_2_O_2_ both in the presence or absence of HI-TOPK-032. As shown in Figure 4B, HI-TOPK-032 reduced the phosphorylation of ERK, which was significantly induced by H_2_O_2_. Given that the ERK signaling pathway, activated by H_2_O_2_-induced oxidative stress, protected cardiomyocytes from apoptosis, we concluded that the ERK signaling pathway might be the downstream mediator of TOPK through which HI-TOPK-032 inhibited the BCL-2/Bax signaling pathway.

### 2.5. TOPK Overexpression Protected H9C2 Cardiomyocytes from Oxidative Stress Injury via the ERK Signaling Pathway

**Figure 5:**
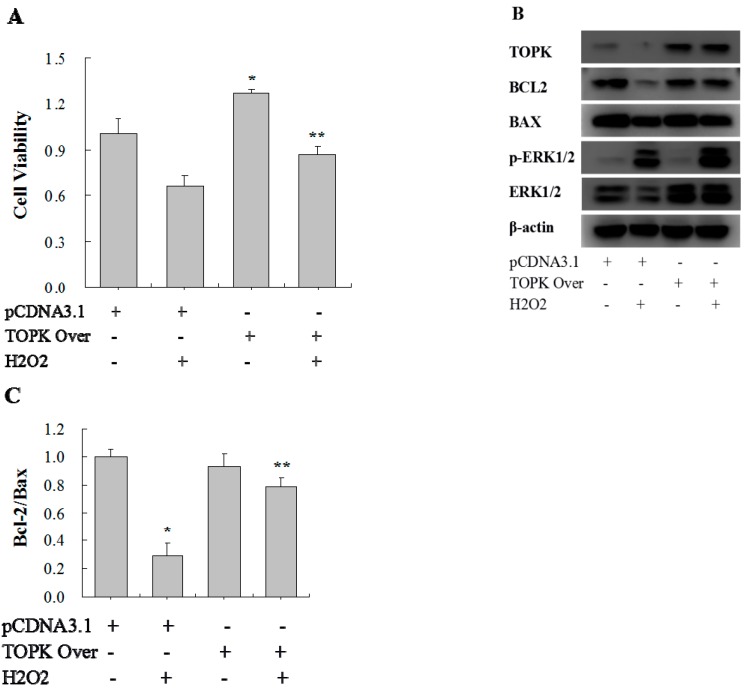
Analysis of the effects of TOPK overexpression on cell viability, apoptosis, and protein expression. H9C2 cardiomyocytes were stimulated with 750 µM of H_2_O_2_ for 1 h after TOPK overexpression plasmid/empty plasmid transfection for 72 h. (**A**) Cell viability as detected by MTS assay; (**B**) Western blot analysis of Bcl-2, Bax, ERK, and p-ERK expression. β-actin was used as a loading control; (**C**) Comparison of the Bcl-2/Bax ratio. * *p* < 0.05 compared with nonstimulated controls with empty plasmid transfection; ** *p* < 0.05 compared with H_2_O_2_-stimulated controls with empty plasmid transfection.

To further understand and validate the effect of TOPK in the injury of H9C2 cardiomyocytes, TOPK cDNA was overexpressed. After transfection with pCDNA3.1(+) (empty plasmid) or pCDNA3.1+TOPK, H9C2 cells were incubated with or without H_2_O_2_ (750 µM) for 1 h to induce oxidative stress injury. Later the cell viability of H9C2 cells was determined by MTS assay. As a result, TOPK overexpression increased both the basal cell viability and the cell viability following oxidative stress injury (Figure 5A). TOPK overexpression also increased the ratio of BCL-2/Bax, which was decreased by H_2_O_2_, and, at the same time, it increased ERK signaling activity, including the total level of ERK and ERK phosphorylation (Figure 5B,C).

## 3. Discussion

In this study, we identified for the first time a function of TOPK protein in myocardial I/R and oxidative stress injury in cardiomyocytes. The novel findings included the following: (1) TOPK was significantly activated by local IPC as assessed by increased phosphorylation of TOPK in the nuclei and resulted in the alleviation of *in vivo* myocardial I/R injury; (2) TOPK was also markedly activated by H_2_O_2_-induced oxidative stress in a time-dependent manner, and the p-TOPK level was increased by 15 min, peaked at 30–60 min, and returned back to the basal level at 120 min after the addition of H_2_O_2_; (3) pharmacological inhibition of TOPK aggravated H_2_O_2_-induced oxidative stress injury in cardiomyocytes, whereas overexpression alleviated it; and (4) mechanistic studies demonstrated that the ERK pathway was downstream of TOPK. Taken together, these results demonstrate that TOPK acted as a novel apoptotic inhibitor, was activated by IPC and H_2_O_2_, and thus contributed to cardioprotection.

IPC, which is accomplished by brief, noninjurious periods of myocardial ischemia and reperfusion, confers protection against I/R injury through endogenous mechanism [20,21]. Similar to IPC, short episodes of I/R during myocardial reperfusion are also associated with a reduction in myocardial infarction size, called ischemic postconditioning (IPostC) [22]. Consistent with previous studies, we showed that three cycles of 5-min ischemia and 5-min reperfusion before I/R was an effective IPC procedure to suppress I/R-induced myocardial injury and demonstrated the cardioprotective role of IPC. Numerous studies have been conducted to explore the underlying mechanisms of IPC with a goal of identifying different pharmacological approaches that would imitate IPC. It has been shown that the cardioprotection by IPC is mediated by activation of survival signaling [23]. TOPK, a novel member of the MAPKK family, is involved in many cellular functions including the promotion of tumor cell proliferation and inhibition of apoptosis [12,24]. Furthermore, a recent study has identified that activation of TOPK pathway is one major mechanism through which IPostC relieves cerebral I/R injury, and the antioxidative effects of TOPK contribute to the neuroprotection of IPostC [25]. In our study, we have demonstrated TOPK protein expression in the myocardium for the first time and observed that its phosphorylation was significantly upregulated by IPC treatment. Therefore, we speculate that TOPK is a novel potential survival signal that could be activated by IPC and mediate its cardioprotective role.

Oxidative stress has been considered as a major mechanism in myocardial I/R injury [26] and is suppressed by IPC to protect against I/R injury [27]. Most studies have reported that IPC actually reduces the overall ROS production in reperfused hearts [28]. On the contrary, it is a surprising observation that ROS generation is actually required for the protective function of both IPC [29] and IPostC [30]. Therefore, ROS play a complex role in myocardial I/R injury and can be both protective and harmful [31]. Superoxide dismutase, N-2-mercaptopropionyl glycine [32], and ascorbic acid [33], which are free radical scavengers, have been shown to negate the protective effect of IPC. Acute administration of vitamin E, which is an antioxidant agent, limited infarction size and preserved the benefits of IPC [34]. Thus, the influence of antioxidants on the *in vivo* effects of IPC and IPostC remains controversial. Because the application of free radical scavengers was limited, exploring the underlying protective endogenous signaling became quite important [35].

In our experiments, only the injurious role of free radicals was involved and H_2_O_2_ treatment was used to imitate the oxidative stress injury during reperfusion. We observed that TOPK was rapidly and transiently activated by H_2_O_2_, consistent with other mitogen-activated protein kinases (MAPKs) in a previous study [6]. HI-TOPK-032, which specifically inhibits TOPK both *in vitro* and *in vivo* and strongly suppresses colon cancer cell growth [18], was used to inhibit TOPK activity in our experiments. This inhibition led to the aggravation of cardiomyocyte injury. In contrast, overexpression of TOPK reversed the reduced cell viability and the BCL-2/Bax activity caused by H_2_O_2_. Therefore, it was concluded that TOPK is a novel potential antioxidative stress regulator, which protected H9C2 cells from H_2_O_2_-induced injury. Moreover, its activation by IPC might further act as a survival signal and appears to be involved in cardioprotection.

The crosstalk between TOPK and Bcl-2/Bax activity in H_2_O_2_-stimulated H9C2 cardiomyocytes was further investigated. Some studies have already been conducted with respect to downstream targets. The ERK pathway has been shown to be an important downstream target of TOPK in the metastasis of prostate cancer cells [36] and in the antioxidative neuroprotection against focal cerebral I/R injury [37]. Additionally, the PI3K/PTEN/AKT pathway has been observed to be involved in the TOPK-mediated promotion of lung cancer cell migration [38] and the proliferation of hepatocellular carcinoma cells [24]. Some additional downstream targets of TOPK are MAPK family members including JNK and p38 [8,39]. Accumulating evidence has shown that the ERK pathway is also involved in the anti-apoptotic effect and cardioprotection against myocardial I/R [40] and oxidative stress injury [6]. Consistent with these previous results, we also observed a significant increase in the ERK and p-ERK levels after TOPK overexpression in H9C2 cells either with or without H_2_O_2_ stimulation, which suggests that ERK is the downstream target of TOPK. This observation was further supported by the fact that the TOPK inhibitor suppressed phosphorylation of ERK.

In conclusion, our study demonstrated for the first time that TOPK mediated a novel survival signal in myocardial I/R injury and was significantly activated by IPC. The anti-oxidative stress effects of TOPK might be achieved by the downstream ERK signaling pathway. However, further studies should be conducted to confirm the role of TOPK in myocardial I/R and the *in vivo* effects of IPC and IPostC, in addition to exploring other related signaling pathways mediating its function.

## 4. Materials and Methods

### 4.1. Ethics Statement

All experimental protocols were approved by the Institutional Animal Care and Use Committee (IACUC) of China Medical University (Shenyang, China) (Project identification code: SCXK-2013-0001). All procedures involving animals in the study were performed in accordance with the ethical standards. Animals used in these experiments were male Sprague-Dawley rats weighing 270–290 g. They were housed under conventional conditions with adequate temperature (25 °C) and a 12-h light/12-h dark cycle with free access to food and water. The rats were anesthetized using sodium pentobarbital (70 mg/kg, i.p.). Every effort was made to minimize the number and suffering of animals in this study.

### 4.2. In Vivo Experimental Protocols

Rats were randomly assigned to one of three groups: sham-operation, I/R with IPC, and I/R without IPC. Myocardial I/R and the IPC procedure were performed as described in a previous study [41]. After rats were anesthetized by pentobarbital with no response to tail pinch, trachea intubation was performed and mechanical ventilation was established. A left thoracotomy was carried out, and a 6-0 silk suture was passed under the left anterior descending coronary artery (LAD) about 2 mm below the left auricle. Then, a slipknot was made around the LAD. An electrocardiogram (ECG) was recorded continuously. Occlusion of the LAD resulted in pallor heart color, reduced cardiac contraction, and ECG changes. Reperfusion was achieved by releasing the ligature and verified by the return of red color, improved cardiac contraction, and ECG recovery. Rats were subjected to 30 min of ischemia and 3 h of reperfusion. IPC was performed with three 5-min cycles of coronary occlusion and 5 min reperfusion. Sham-operated rats underwent the same surgical procedures except that the suture around the LAD was not ligated. Myocardial infarct size was detected by Evans blue-TTC double staining in the I/R and IPC+I/R groups (*n* = 6 per group). The myocardium at the ischemic area was collected from the three groups (sham-operation, I/R, and IPC+I/R; *n* = 6 per group) and divided into three parts: one for the terminal deoxynucleotidyl transferase-mediated dUTP nick end labeling (TUNEL) assay, hematoxylin and eosin (HE) staining, and immunohistochemical staining; one for western blot analysis; and another for PCR analysis.

### 4.3. Cell Culture and in Vitro Experimental Protocols

The H9C2 rat ventricular cells were obtained from the Cell Bank of the Chinese Academy of Sciences (Shanghai, China) and cultured in Dulbecco’s modified Eagle’s medium supplemented with 10% fetal bovine serum, 100 mg/mL streptomycin, and 100 IU/mL penicillin at 37 °C in a 5% CO_2_ humidified incubator. In all experiments, cells were subjected to serum starvation for 24 h before treatment. To initiate oxidative stress, H9C2 cells were exposed to H_2_O_2_ (0–1000 µM) for the indicated times. To explore the function of TOPK, its inhibition by the inhibitor HI-TOPK-032 (Sigma-Aldrich, St. Louis, MO, USA) and TOPK gene overexpression were both conducted in our study. DMSO was used to dissolve the TOPK inhibitor HI-TOPK-032, and an equivalent concentration of DMSO, as used for the maximal HI-TOPK-032 treatment, was used to treat cells as a solvent control. For TOPK overexpression, the H9C2 cardiomyocytes were transfected at 50% confluence with pCDNA3.1(+) (empty plasmid) or pCDNA3.1+TOPK (rat TOPK overexpression recombinant plasmid), provided by Wuhan Genecreate Biological Engineering Co., Ltd. First, the medium was replaced with OPTI-MEM (Gibco, Carlsbad, CA, USA), and Lipofectamine 2000 (Invitrogen, Carlsbad, CA, USA) was then used as the media for transfection according to the instructions of manufacturer. Five hours later, the medium was replaced with the growth medium. The cells were cultured for 72 h after transfection, and each group (empty plasmid group and TOPK overexpression group) was assigned to two subgroups and treated or not treated with H_2_O_2_. All variables were tested in three independent cultures for each experiment.

### 4.4. Determination of Myocardial Infarct Size

Myocardial infarct size was determined by Evans blue-TTC double staining. Briefly, the suture around the LAD was retightened at the end of reperfusion, and 2% Evans blue dye was injected into the left ventricular cavity. Then, the heart was quickly excised, frozen, and sliced transversely into five sections, each of which was incubated in 1% TTC for 15 min at 37 °C and then digitally photographed. The Evan’s blue-negative stained area (area at risk, AAR), TTC-negative staining area (infarcted area, IA), and the total left ventricular area (LV) were measured using the ImageJ software (NIH Image, Bethesda, MD, USA). Infarction size was expressed as the percentage of IA over AAR (IA/AAR) and the size of AAR was expressed as the percentage of AAR over total LV area (AAR/LV).

### 4.5. In-Situ Detection of Apoptosis in Heart Tissue

The effects of I/R and IPC on myocardial cell apoptosis were quantified using the TUNEL assay with a kit (KeyGEN, Nanjing, China) according to the manufacturer’s instructions. Cells were observed in three fields, and the nuclei with brown color were considered as positively stained cells. The percentage of myocardial cell apoptosis was calculated as the ratio of positive cells to the total cells.

### 4.6. HE and Immunohistochemical Staining

Specimens were fixed in 4% paraformaldehyde, embedded in paraffin, serially sectioned, and stained with HE. The immunohistochemical staining of paraffin sections was performed according to the standard procedures. In brief, the tissue sections were incubated with primary antibodies to p-TOPK Thr9 (Abcam, Cambridge, UK) and TOPK (Cell Signaling Technology, Danvers, MA, USA) at dilutions of 1:100 at 4 °C overnight. After incubation with a biotinylated secondary antibody for 10 min, the sections were stained with diaminobenzidine and counterstained with hematoxylin. Finally, images were captured using a microscope (Leica DM4000 B LED, Leica Microsystems, Heidelberg, Germany).

### 4.7. Quantification of Cell Viability by MTS Test

Cell viability was evaluated with the MTS assay. Cells were incubated with CellTiter 96^®^ AQueous One Solution Cell Proliferation Assay (Promega, Madison, WI, USA) for 2 h at 37 °C, and the absorbance at 490 nm was recorded using the PowerWave XS Microplate Reader (BioTek Instruments, Inc., Winooski, VT, USA).

### 4.8. Immunofluorescence Analysis

Cell immunofluorescence was performed according to well-established procedures. In brief, cells on coverslips were gently washed with phosphate-buffered saline (PBS), fixed with 3.7% paraformaldehyde, and permeabilized with 1% Triton X-100. After the cells were incubated with the primary antibody against p-TOPK Thr9 (Abcam, 1:100), the corresponding alexa 546-conjugated fluorescent secondary antibody (Life Technologies, Carlsbad, CA, USA, 1:150) was added. The nuclei were stained and visualized with DAPI in the dark. Finally, fluorescent images were visualized and captured using a fluorescence microscope (Leica DM4000 B LED, Leica Microsystems, Heidelberg, Germany).

### 4.9. Real-Time Quantitative PCR

Total RNA was isolated from the myocardial tissues with TRIzol Reagent (Invitrogen) and purified using Qiagen’s RNeasy Total RNA Isolation Kit. Real-time quantitative PCR (RT-PCR) was performed using the PrimeScript^TM^ RT Reagent Kit (TaKaRa, Dalian, China) and SYBR^®^ Premix Ex TaqTM II (TaKaRa) by the Applied Biosystems StepOnePlus^TM^ Real-Time PCR System (Life Technologies). The PCR primers used were as follows: rat GAPDH (GenBank Accession No. NM017008), forward 5’-CACTGAGGACCAGGTTGTCT-3’ and reverse 5’-TCCACCACCCTGTTGCTGTA-3’; rat TOPK (GenBank Accession No. NM001079937), forward 5’-TTGCTATGGAGTATGGAGGTG-3’ and reverse 5’-GATACTTTAGCCCTCTTGCCA-3’. All of the primers were synthesized by Wuhan Genecreate Biological Engineering Co., Ltd. (Wuhan, China). The real-time PCR data were expressed as *C*t values, defined as the crossing threshold of PCR using the Applied Biosystems StepOnePlus^TM^ Real-Time PCR System Data Analysis Software. TOPK mRNA expression in each sample was normalized to corresponding GAPDH expression and calculated as 2^−ΔΔ*C*t^.

### 4.10. Western Blot Analysis

Tissues and H9C2 cells were lysed in a cold radio-immunoprecipitation assay (RIPA) lysis buffer (Beyotime Biotechnology, Nantong, China) according to standard protocols, and the protein concentrations of lysates were determined using the Pierce BCA Protein Assay Kit (Thermo Scientific, Rockford, IL, USA). Equal quantities of proteins were separated by 12% sodium dodecyl sulfate (SDS)-polyacrylamide gel electrophoresis (PAGE). The proteins in the gels were transferred to polyvinylidene difluoride membranes, blocked, and then incubated with the following primary antibodies (1:1000) overnight at 4 °C: p-TOPK Thr9 (Abcam), TOPK, p-ERK1/2 Thr202/Tyr204, ERK1/2 (Cell Signaling Technology), BAX, BCL-2, and β-actin (Proteintech, Wuhan, China). The positive signals were detected after incubation with the corresponding horseradish peroxidase-conjugated secondary antibodies (Proteintech) for 2 h at room temperature. Protein bands were detected using the enhanced chemiluminescence (ECL) western blotting substrate (Thermo Scientific), and the intensity was quantified using ImageJ 1.47 software.

### 4.11. Statistical Analysis

The experimental results were reported as mean ± SD and analyzed with a Student’s unpaired t-test or one-way analysis of variance (ANOVA). All statistical analyses were performed using SPSS 17.0 statistical software (SPSS, Inc, Chicago, IL, USA). *p* < 0.05 was considered to be statistically significant.

## Abbreviations

AMI: acute myocardial infarction;
DMSO: dimethyl sulfoxide;
ECG: electrocardiogram;
ECL: enhanced chemiluminescence;
ERK: extracellular signal-regulated kinases;
H2O2: hydrogen peroxide;
HE: hematoxylin and eosin;
I/R: ischemia/reperfusion;
IPC: ischemic preconditioning;
LAD: left anterior descending coronary artery;
LV: left ventricular;
MAPK: mitogen-activated protein kinase;
MAPKK: mitogen-activated protein kinase kinase;
ROS: reactive oxygen species;
TOPK: T-LAK-cell-originated protein kinase;
TUNEL: terminal deoxynucleotidyl transferase-mediated dUTP nick end labeling.
